# Tuning Nanostructure of Gels: From Structural and Functional Controls to Food Applications

**DOI:** 10.3390/gels11080620

**Published:** 2025-08-08

**Authors:** Tangyu Yang, Lin Cao, Junnan Song, Andre G. Skirtach

**Affiliations:** Nano-Biotechnology Group, Faculty of Bioscience Engineering, Ghent University, 9000 Ghent, Belgium; tangyu.yang@ugent.be (T.Y.); lin.cao@ugent.be (L.C.); junnan.song@ugent.be (J.S.)

**Keywords:** nanostructure, gelation, biopolymers, rheology, controlled release, texture, food structure design, 3D food printing, functional foods

## Abstract

Various gels are integral for the food industry, providing unique textural and mechanical properties essential for the quality and functions of products. These properties are fundamentally governed by the gels’ nanostructural organization. This review investigates the mechanisms of nanostructure formation in food gels, the methods for their characterization and control, and how precise tuning of these nanostructures enables targeted food applications. We examine the role of various building blocks, including biopolymers, lipids, and particles, and the gelation mechanisms leading to specific nanostructures. Advanced techniques (e.g., microscopy, scattering, spectroscopy, and rheology) are discussed for their insights into nano-/microstructures. Strategies for tuning nanostructures through chemical composition adjustments (e.g., concentration, pH, ionic strength) and physical processing controls (e.g., temperature, shear, ultrasound) are presented. Incorporating nanostructures like nanoparticles and nanofibers to enhance gel properties is also explored. The review links these nanostructures to key functional properties, including mechanical strength, water-holding capacity, optical characteristics, and bioactive delivery. By manipulating nanostructures, products can achieve tailored textures, improved stability, and controlled nutrient release. Applications enabled by nanostructure tuning include tailored sensory experiences, fat reduction, innovative food structures, and smart packaging solutions. Although significant progress has been made, precise structural control and a comprehensive understanding of complex nanoscale interactions in food gels remain challenging. This review underscores the importance of nanostructure tuning in food gels, highlighting its potential to drive future research that unlocks innovative, functional food products.

## 1. Introduction

Food gels are soft materials characterized by a continuous three-dimensional (3D) network that encapsulates significant volumes of liquids, such as water or oil, resulting in a viscoelastic system that exhibits properties intermediate between solids and liquids [1,2]. These networks, typically formed from biopolymers such as proteins [3], polysaccharides [4], or a combination of other biopolymers [5,6,7], are classified as hydrogels [6], oleogels [8], or bigels [9], depending on the entrapped liquid. The 3D network is critical for immobilizing liquids, which underpins the gels’ distinctive textural and mechanical properties, including firmness, elasticity, and water-holding capacity (WHC) [10]. These attributes significantly influence the texture, mouthfeel, and sensory appeal of a wide range of food products, such as yogurt, cheese, jellies, sauces, processed meats, and plant-based analogs [11,12]. Additionally, food gels enhance structural stability, prevent phase separation, extend shelf-life, and serve as carriers for bioactive compounds, enabling controlled release and improved stability of nutrients, flavors, and functional ingredients [5,10].

The adaptability of food gels arises from their tunable properties, which allow for the design of products with customized textures, ranging from smooth and soft to firm and elastic [13,14], and tailored to meet specific dietary needs, such as reduced-fat formulations or foods for individuals with swallowing difficulties [15]. This versatility supports their use in traditional foods like tofu, cheese, and jellies [2], as well as in innovative applications, including plant-based analogs, 3D-printed foods, and edible coatings [16].

The functionality of food gels is fundamentally governed by their nanoscale structural organization [17]. The arrangement and interactions of molecular building blocks (e.g., proteins [18], nanoparticles or nanocrystals [19], and low-molecular-weight gelators [20,21]) directly determine macroscopic properties like texture, mechanical strength, and performance [15]. For instance, in protein-based gels, nanoscale gelation and aggregation processes form network structures that encapsulate bioactive compounds, thereby affecting texture, stability, and nutritional functionality [18,22]. Likewise, the self-assembly of nanoparticles or nanocrystals into interconnected networks enables the transfer of size-dependent properties to the macroscale, with nanoscale connectivity influencing mechanical and transport behaviors [19,23,24]. Hierarchical self-assembly of molecular gelators into defined nanostructures enables the creation of tunable stimuli-responsive, self-healing properties gels [20,25].

Rational design of food gels depends on manipulating this nanoscale organization. By modifying building blocks through chemical functionalization, adjusting assembly conditions, or using specific linkers [26], researchers can control network connectivity, porosity, and mechanical properties to achieve targeted functionalities, such as enhanced toughness or specific release profiles [17,18]. Understanding the relationships between nanostructure, structure, and properties is thus essential for developing advanced food gels with precisely engineered textures, stability, and functional benefits.

In food gels, the nanostructure-function relationship is driven by the self-assembly of biopolymers or colloids into 3D networks. The nanoscale architecture—determined by such factors as type, density, connectivity, and intermolecular interactions (e.g., hydrogen bonding, electrostatic forces)—directly governs mechanical strength, liquid retention, and textural qualities [15,20]. By fine-tuning parameters like nanofibril morphology, concentration, and assembly conditions, scientists can customize gel properties, including elasticity, breakdown behavior, and responsiveness to external stimuli [27,28,29,30]. The stability and functionality of these gels rely on both covalent and non-covalent bonds between nano-building blocks, such as nanofibers or protein–polysaccharide complexes [30,31]. Techniques such as ionic gelation, composite systems [32], and responsive supramolecular designs [29] enable the engineering of sophisticated food gels with properties optimized for advancements in nutrition, sensory experience, and sustainability.

Spanning the disciplines of food science, materials science, colloid chemistry, and rheology, this review consolidates contemporary knowledge on the mechanisms governing nanostructure formation in food gels, the methodologies for characterizing and controlling these structures, the correlation between nanostructure and functional properties, and the ways in which precise structural control can be harnessed for targeted applications. Through this interdisciplinary lens, it aims to illustrate how tuning nanostructures can catalyze innovation in food gels’ development, thereby addressing consumer needs for quality, health, and sustainability.

## 2. Building Blocks and Gelation Mechanisms

### 2.1. Structuring Agents

**Figure 1:**
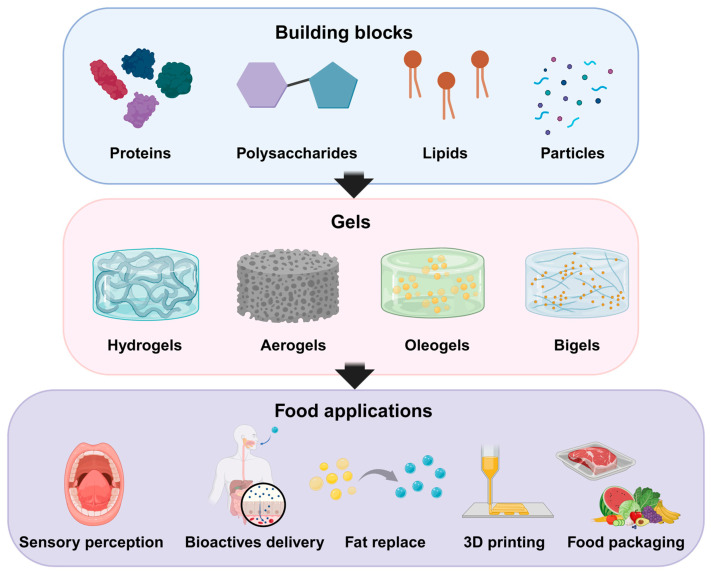
Overview of building steps and structures of food gels, including hydrogels, aerogels, oleogels, and bigels, and their application fields. Created in BioRender. group, n. (2025) https://BioRender.com/orxhurf (accessed on 10 July 2025).

Food gels are primarily structured from biopolymers, with proteins and polysaccharides serving as the main building blocks, as shown in Figure 1 [33,34].

#### 2.1.1. Proteins

Proteins, including globular types (such as whey, soy, and egg proteins), fibrous proteins (like gelatin and collagen), and casein micelles, form gels mainly through heat-induced denaturation and aggregation or by cooling, as seen with gelatin [22,35]. Additionally, peptides, especially those capable of self-assembly, act as adaptable building blocks in food gels by organizing into nanostructures like fibrils, nanotubes, and micelles [36]. The gelation process for proteins is influenced by factors such as pH, ionic strength, and temperature, which affect their intermolecular interactions and ultimately the texture and microstructure of the gel network [3,18]. Furthermore, the interactions between different proteins (e.g., globular proteins, casein, and gelatin) during heating or cooling significantly affect gel stiffness and microstructure, with scenarios including independent aggregation, co-aggregation, and phase separation being key considerations [37,38]. This is exemplified in an ultrasound-treated soy protein isolate/egg white protein (SPI-EWP) composite gel [39] where accelerated co-aggregation kinetics (due to reduced electrostatic repulsion and exposed sulfhydryl groups) form dense yet porous networks, enhancing gel stiffness in SPI-dominated systems but increasing heterogeneity in EWP-rich gels.

#### 2.1.2. Polysaccharides

Polysaccharides, another essential group, include helix-forming types (κ-carrageenan, gellan), charged varieties (alginate, pectin, xanthan), and neutral forms (starch, cellulose derivatives) [4]. Their gelation depends on hydration, ionic interactions, and molecular entanglement, and they are often used in combination with proteins to create composite gels with tailored properties [4,40]. Recent advances include the creation of smart hydrogels with antimicrobial and barrier properties, and the use of oleogels and emulsion gels as healthier fat substitutes [41]. Additionally, new drying technologies [42] and composite gel designs are expanding applications in food structuring, even biomedical fields, though challenges remain in optimizing gel properties and integrating sustainable raw materials [43].

The interplay between proteins and polysaccharides allows for the design of gels with diverse structures and functionalities, making them highly valuable in food applications for texture modification, stability, and delivery of bioactive substances [44]. For example, rice starch and soybean protein exhibit antagonistic interactions in composite gels, resulting in phase-separated networks with reduced gel strength and the International Dysphagia Diet Standardization Initiative (IDDSI) Level 5 suitability, enabling softer texture-modified foods for moderate dysphagia patients when blended at a 50:50 ratio [45]. Such antagonistic or synergistic effects are highly dependent on the specific biopolymer pairings and their microstructural compatibility, highlighting the need for continued research into structure–function relationships.

#### 2.1.3. Lipids

Lipid-based food gels serve as essential carriers for lipophilic ingredients, utilizing either crystalline networks (oleogels/organogels [10,46]) or emulsified structures (emulsion gels [47,48]). Crystalline networks form through self-assembling gelators that trap oil in solid-like matrices, offering controlled release and stability for fat-soluble compounds [46]. Emulsion gels incorporate oil droplets within aqueous or lipid phases stabilized by proteins, polysaccharides, or particles (e.g., Pickering emulsions [49]), enabling encapsulation of both hydrophilic and lipophilic components [23]. Both systems can improve stability, bioavailability, and release kinetics of bioactives, with these properties influenced by factors such as gelator type, crystalline structure, droplet size, and stabilizer selection. Emulsion gels also offer additional benefits for fat replacement and texture modification in functional foods [50,51]. These tunable structures provide versatile solutions for optimizing sensory properties and health-oriented delivery systems.

Recent research has focused on developing healthier fat alternatives, improving functional properties, and expanding applications in food technology [46,48]. Glyceryl monostearate-based oleogels have been shown to form solid-like gels with sunflower and high oleic sunflower oils, with the oil type influencing gel network formation and crystal morphology, which can be tailored for improved nutritional profiles in food products [52]. Moreover, surfactant-free, plant-based double emulsion gels (O/W/O and W/O/W) have been engineered as low-calorie fat analogs, offering tunable oral perception, inhibition of lipid digestion, and effective co-delivery of bioactives like lycopene and epigallocatechin gallate (EGCG), with O/W/O gels mimicking butter’s texture and both types showing anti-inflammatory activity [53]. Comparative studies of structuring agents such as wax crystals, hydrophilic cellulose derivatives, and emulsion droplets reveal distinct rheological and thermal behaviors, guiding the selection of gel systems for specific food applications [54]. The field is also seeing rapid innovation in delivery systems, with trends moving toward complex structures like Pickering emulsions, bigels, and multiple emulsions, which improve bioactive delivery, sensory experience, and enable reduced-fat formulations [55].

#### 2.1.4. Particles

Particles serve as building blocks in food gels because they influence their texture, stability, encapsulation, and release properties (Figure 1) [56,57]. Their incorporation into gels leads to the formation of the so-called hybrid structures [58]. Particles such as protein-based fillers can increase the elastic modulus, mechanical properties, and firmness of food gels, depending on their size, concentration, and interaction with the gel matrix [59,60]. However, gels can also be filled with emulsions [61], whose concentration can be used to tune mechanical properties. Further, gel particles can act as stabilizers in Pickering emulsion gels, forming a dense layer around oil droplets and preventing coalescence [62]. This enhances the stability and layer-by-layer interfacial structure of emulsions, which is important for products like dressings and spreads. Recent advances in particle-filled food gels have focused on enhancing mechanical properties, developing novel structures, and expanding applications in food technology [63,64]. Innovative strategies such as particle/fiber reinforcement, double-network formation, dual cross-linking, and freeze-thaw cycles have been shown to significantly improve gel strength and resilience, depending on the type of filler, gel matrix, and their interactions [63]. For example, incorporating glass microspheres into whey protein isolate/xanthan gum gels increased the elastic modulus, with effects influenced by filler size, polydispersity, and ionic strength [59]. Thereinto, glass microspheres and similar rigid particles are frequently used as model fillers in research to investigate the structural and mechanical properties of composite food gels, though they are not intended for direct consumption. Novel emulsion microgel particles, created by combining gelatinized native starch and octenyl succinic anhydride starch-stabilized emulsion droplets, can be tailored for the delivery of lipophilic molecules in food systems [65]. Additionally, the development of thermo-responsive double-interpenetrating colloidal-particle networks, such as those combining silica and lipid particles, has resulted in gels with enhanced stiffness and resilience, suitable for 3D-printed food constructs [13]. Advances in microrheology and particle tracking have further enabled detailed mapping of local viscoelastic properties and structural heterogeneities in food gels [66]. These developments collectively highlight the growing potential of particle-filled food gels for applications in personalized nutrition, fat replacement, nutrient delivery, and 3D food printing [1,15].

### 2.2. Gelation Mechanisms and Nanostructure Genesis

**Figure 2:**
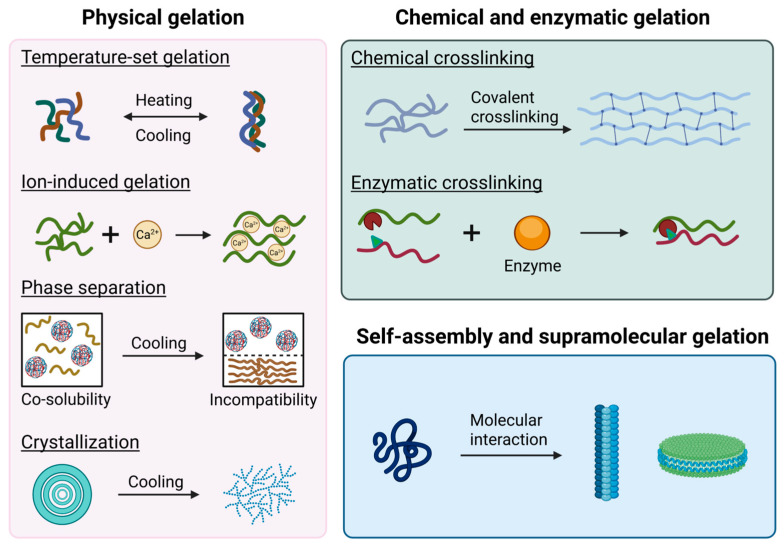
Summary diagram of the gelation mechanism, including: Temperature-set gelation: temperature-induced gelatin gels; Ion-induced gelation: Ca^2+^-induced alginate gels forming egg-box structure; Phase separation: phase separation and gelation in konjac glucomannan and gelatin system (reproduced from [70] with permission from Elsevier); Crystallization: temperature-induced starch retrogradation to form gels; Chemical cross-linking: polymers chains crosslinked by cross-linking agent; Enzymatic cross-linking: polymers chains crosslinked by enzyme; Self-assembly and supramolecular gelation: peptides or low-molecular-weight gelators form supramolecular architectures through molecular interactions.

The nanostructure and functional properties of food gels are determined by their gelation mechanism, whether physical (e.g., heat/cool setting, ion-induced, phase separation, crystallization), chemical/enzymatic (e.g., covalent cross-linking via enzymes like transglutaminase), or supramolecular self-assembly (e.g., peptides, sugar derivatives [20]). The gelation mechanisms were summarized and illustrated in Figure 2.

#### 2.2.1. Physical Gelation

**Figure 3:**
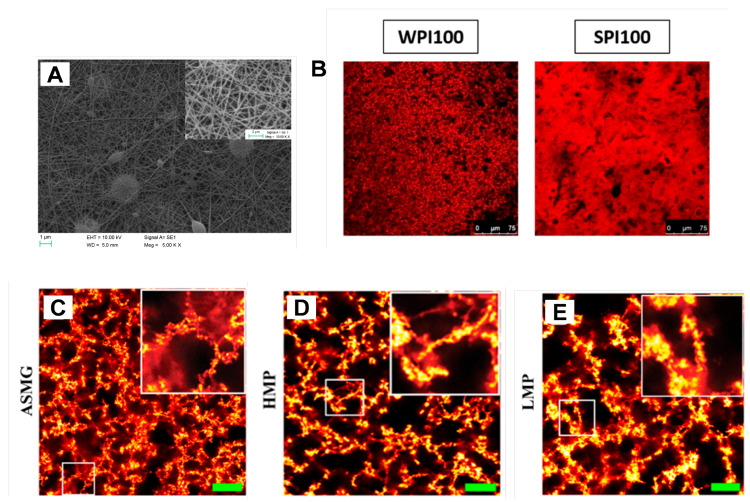
(**A**) SEM image of gelatin/chitosan/3-phenylacetic acid nanofiber film (adapted from [76] with permission from Elsevier). (**B**) Micrographs of whey protein isolate (WPI) and soy protein isolate (SPI) gels obtained through confocal laser scanning microscopy (adapted from [77]). High-resolution STED microscopy images of acidified fresh skimmed-milk gels (ASMG) with and without pectin: (**C**) The control gel without pectin; (**D**) Gels with high-methoxy pectin (HMP) (AMD 783); (**E**) Gels with low-methoxy pectin (LMP) (PSY 200) (adapted from [78] with permission from Elsevier).

- Heat/Cool-Driven Conformational Transitions. Heat-driven conformational transition occurs when heating unfolds proteins or specific polysaccharides (like konjac glucomannan), promoting aggregation and network formation through hydrophobic interactions, hydrogen bonds, and sometimes disulfide bonds, often facilitated by additional factors like alkali treatment; conversely [18,35]. Cool-driven conformational transition occurs upon cooling, allowing polymers such as gelatin or amylose in starches to reassociate and form stable junction zones and networks, where the cooling process critically determines the resulting gel’s texture and firmness [67].
- Ion-Induced Gelation. Addition of ions (e.g., Ca^2+^) to protein or polysaccharide systems (such as alginate or pectin) promotes conformational changes (e.g., α-helix to β-sheet in proteins), enhances hydrophobic interactions, and leads to the formation of stable, ordered 3D networks, as shown in Figure 3A. Excessive ion concentration also causes irregular aggregates and coarser structures [68].
- Phase Separation. Mixing incompatible biopolymers (e.g., gelatin and polysaccharides) can induce phase separation, resulting in hierarchical or multi-domain microstructures within the gels [69,70].
- Crystallization. In starch and polysaccharide gels, particularly starch-based systems, crystallization is a key mechanism where linear polymer chains (like amylose) align and associate during cooling, forming stable crystalline junction zones that significantly reinforce the gel network structure; the degree of this crystallization critically determines the gel’s firmness, stability, and overall textural quality in the final product [10,67].

#### 2.2.2. Chemical and Enzymatic Gelation

- Chemical Gelation. Food gels are mainly stabilized by non-covalent interactions such as hydrogen bonds, electrostatic forces, Van der Waals forces, and hydrophobic interactions, which enable the formation of three-dimensional networks without the need for covalent cross-linking [33,34]. However, disulfide bonds serve as an important exception, providing covalent cross-linking that enhances stability and rigidity in protein gels [15,71]. The resulting gel network’s strength and structure are strongly influenced by factors including temperature, pH, ionic strength, the concentration of gelling agents, and the presence of small molecules such as sugars, acids, and salts [33,67,72].
- Enzymatic Gelation. The formation of covalent bonds or modify polymer structures in enzymatic gelation are catalyzed by enzymes [73], where transglutaminase and specific proteases induce protein gelation by cross-linking molecules, creating networks with unique textures [73,74], while in polysaccharide systems (e.g., carrageenan, agar, alginate), enzymes like epimerases, desulfatases, and lyases alter the carbohydrate backbone to tailor gel characteristics [74].

#### 2.2.3. Self-Assembly and Supramolecular Gelation

Self-assembling peptides and low-molecular-weight gelators (including sugar derivatives) form nanofibers, nanoparticles, or hierarchical supramolecular architectures through non-covalent interactions [29,36]. Supramolecular gels exhibit diverse nanostructures (fibrils, strands, platelets) that can be modulated for specific functions, such as encapsulation or stimuli-responsiveness [20,36].

## 3. Characterization of Nano-/Microstructures in Food Gels

### 3.1. Microscopy Techniques

Cryo-scanning electron microscopy (Cryo-SEM), transmission electron microscopy (TEM), and atomic force microscopy (AFM) have provided 3D images of various food gels, identifying unique structural features such as fiber networks in heat-induced milk gels and thick chains in acid–heat gels (Figure 3A) [75,76]. Confocal Raman microscopy has mapped protein and water distribution in mixed whey/soy gels, detecting differences in hydration and the presence of residual lipids, with soy-rich regions showing greater auto-fluorescence (Figure 3B) [77]. Stimulated emission depletion (STED) microscopy and fluorescence lifetime imaging microscopy (FLIM) have enabled detailed visualization of protein networks and molecular dynamics and interactions in plant-dairy gels, showing that pectin incorporation increases both the thickness of protein networks and the size of voids, resulting in a more porous and cohesive gel structure (Figure 3C) [78]. STED microscopy, combined with quantitative image analysis, has distinguished between different protein aggregates in acidified milk gels, revealing that nano-particulate whey protein forms larger, more connected aggregates, which correlates with higher gel strength [79]. Light microscopy (LM) as well as polarized light microscopy (PLM), especially with iodine staining, remains a valuable technique for differentiating starch components and monitoring gelatinization, offering more detailed supramolecular insights than SEM [80,81]. Additionally, advanced image analysis and deep learning applied to microscopy images have enabled high-throughput, accurate quantification of starch gelatinization and granule swelling, achieving up to 95% accuracy in identifying birefringence loss during heating [82].

### 3.2. Scattering Techniques

Scattering techniques such as small-angle X-ray scattering (SAXS), small-angle neutron scattering (SANS), and ultra-small-angle neutron scattering (USANS) are powerful non-destructive tools for providing insights into junction zone structures, network density, and persistence length [83,84,85]. These methods enable real-time, in situ monitoring of structural changes during processes like simulated gastric digestion, revealing, for example, that gel firmness and elasticity influence disintegration behavior and digestibility [86]. Additionally, it is confirmed that physical disruption of gel particles at the macroscale does not significantly alter their internal nano- or microstructure, validating the use of blended samples for scattering analysis [87]. Scattering techniques have also elucidated complex hierarchical assembly mechanisms, such as the two-step network formation in nanodiamond hydrogels, and have been combined with imaging to provide comprehensive multi-scale characterization of supramolecular and low-molecular-weight gels [88,89].

### 3.3. Spectroscopy

Fourier-Transform Infrared Spectroscopy (FTIR), as a part of vibrational spectroscopy techniques, provides information on secondary structure and cross-linking, which is vital for understanding the formation and stability of protein-based and polysaccharide-based nanostructures [90]. For example, when combined with chemometric methods, FTIR effectively distinguishes gelatin sources by identifying unique spectral signatures in the amide and fingerprint regions, enabling rapid and robust authentication of bovine, porcine, and fish gelatins [91,92]. Another vibrational spectroscopy technique, Raman spectroscopy, detects vibrations of atoms in molecules but is better suited to samples containing an aqueous solution [93]. Nuclear magnetic resonance (NMR) provides high-resolution structural information on food gels, allowing for the identification of detailed analysis of composition, sequence, and substitution patterns [84]. UV/Vis also enables characterization of nano-/microstructures in food gels, such as tracking nanoparticle migration and separation in hydrogels, identifying specific particle morphologies via absorption peaks, and quantifying particle concentrations within gel matrices, as demonstrated by in situ fiber-based systems and studies on nanocomposite films and microgels [94,95]. Other spectroscopic techniques, like spectrophotometry, spectrofluorimetry, and inductively coupled plasma atomic emission spectroscopy (ICP-AES), offer high sensitivity and selectivity for detecting trace components and monitoring real-time changes in food gels, as demonstrated in the quantification of trace As(III) with low detection limits and wide linear ranges [31,96].

### 3.4. Rheology and Microrheology

**Figure 4:**
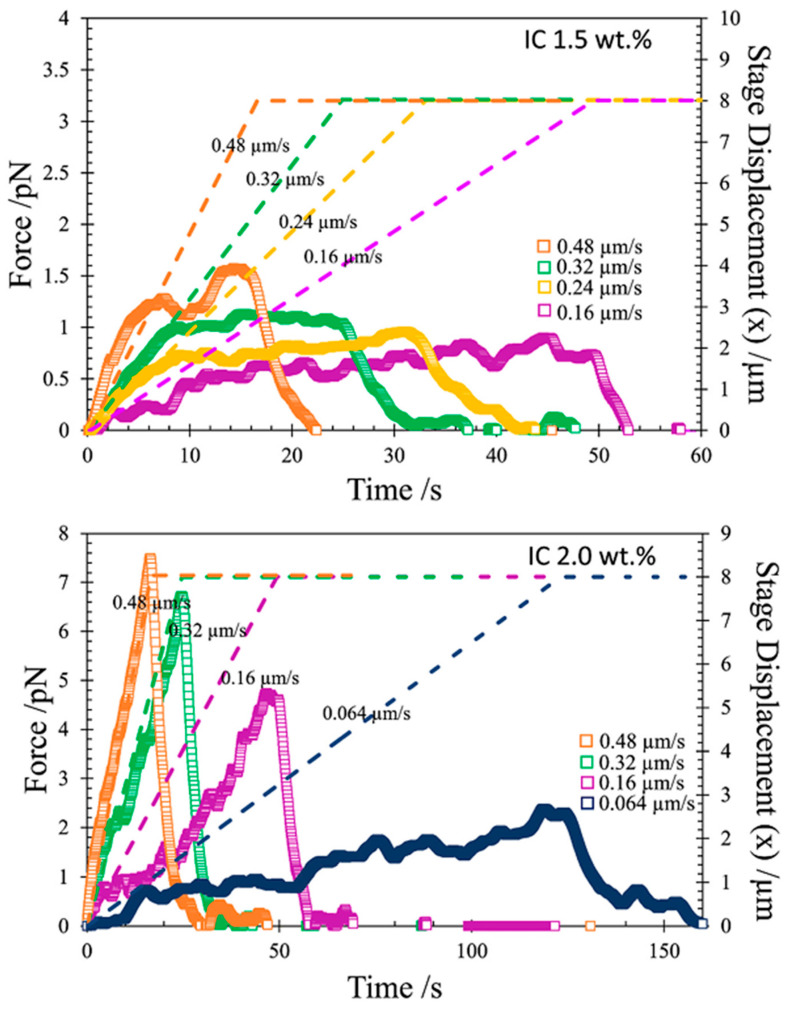
Mesoscale rheology of iota–carrageenan (IC) gels at different strain rates using optical tweezers (OT). The measured force response (symbols) as a function of stage movement at different speeds. The dashed line represents the stage displacement (adapted from [100] with permission from Elsevier).

Traditional rheology provides quantitative insights into the network assembly and viscoelastic behavior of hydrogels formed from various biopolymers, revealing how factors like cross-linking and chain architecture influence properties such as modulus, shear behavior, and thixotropy, which are critical for applications like 3D printing and ingredient delivery in foods [97,98]. Microrheology, using optical tweezers and dynamic light scattering, enables probing of local viscoelastic properties and spatial heterogeneity at the micro- and nanoscale, which is not accessible by bulk rheology [66,99]. For example, microrheological studies of iota–carrageenan gels revealed a concentration-dependent network structure, with increased entanglement density at higher concentrations limiting structural rearrangement and yielding behavior (Figure 4) [100]. In colloidal rod systems, microrheology and bulk rheology together showed that microstructure remains unchanged with increasing colloid concentration, but sol–gel transitions occur with depletant addition, highlighting the complementary nature of these methods for mapping gelation phase diagrams [101]. Additionally, microrheology can detect nanoscopic interfacial viscoelasticity, as seen in poly(ethylene oxide) hydrogels, where deviations between macro- and microrheological measurements revealed the presence of a viscoelastic interfacial shell around tracer particles [102].

### 3.5. Technical Challenges

Characterizing nano-/microstructures in food gels presents several key challenges, including the complexity and heterogeneity of gel networks, the need for advanced imaging and analytical techniques, and the sensitivity of gel structures to environmental and sample manipulation. The intricate interactions between biopolymer building blocks, such as proteins and polysaccharides, require precise quantification at both molecular and network levels, often necessitating the integration of super-resolution microscopy, fluorescence lifetime imaging, and nanomechanical methods to capture spatial and dynamic variations within gels.

## 4. Strategies for Tuning Nanostructure of Gels

### 4.1. Chemical Composition

The formulation of food gels (e.g., the type and concentration of building blocks and other additives, pH, and ionic strength) has a major impact on their nanostructure, gelation behavior, and functional properties. The common strategies for tuning nanostructures of gels, including mechanisms and impacts, are summarized in Table 1.

#### 4.1.1. Concentration

**Figure 5:**
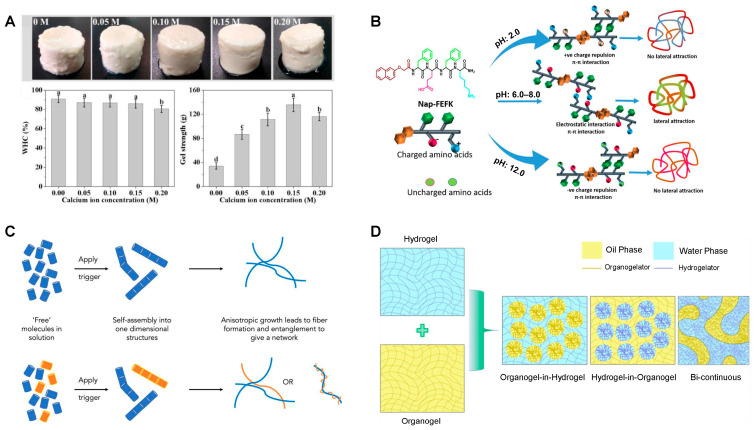
(**A**) Visual appearance, WHC, and gel strength of cellulose nanocrystals-whey protein isolate composite gels with different concentrations of Ca^2+^ (adapted from [68] with permission from Elsevier). Different lowercase letters (a–d) indicate significant differences among treatments (*p* < 0.05). (**B**) Chemical structure of the ionic complementary sequence, naphthoxyacetic acid-FEFK peptide, and schematic representation of the self-assembling behavior of the designed peptide sequence depending on the pH of the system (adapted with permission from [110]. Copyright 2020 American Chemical Society). (**C**) Schematic description of a low-molecular-weight gel assembling to form fibrous structures that entangle to form a network: Single-component and two-component systems (adapted with permission from [111]. Copyright 2022 American Chemical Society). (**D**) Different conventional bigels obtained by varying organogel/hydrogel ratio (adapted from [112] with permission from Elsevier).

Adjusting the concentration of key components such as nanoparticles, proteins, oils, or ions efficiently tunes the nanostructure and functional properties of food gels [15]. For example, increasing concentrations of cellulose nanocrystals (CNC) in whey protein isolate gels (up to 1.0% *w*/*v*) enhances WHC, gel strength, viscoelasticity, and thermal stability by promoting protein unfolding, cross-linking, and the formation of compact, homogeneous gel networks; however, excessive CNC can lead to agglomeration and moisture absorption effects [103]. Similarly, raising calcium ion concentration in composite gels or nanocellulose gels strengthens the gel network and viscoelastic properties by facilitating cross-linking, but too high a concentration (e.g., 0.20 M Ca^2+^) can cause irregular aggregation and reduced gel quality (Figure 5A) [68,104]. For Pickering emulsions stabilized by gelatin nanoparticles, higher nanoparticle concentrations (0.3–2.0 wt.%) result in smaller droplet sizes, increased viscosity, and more compact network structures, with optimal stability and viscoelasticity achieved at 1.5–2.0 wt.% [105]. In protein-based emulsion gels, increasing oil concentration (up to 20% soybean oil) improves gel strength, water-holding, and network formation, but further increases can reduce these benefits [106]. Nanofiber cellulose and faba bean protein isolate concentrations also modulate gel hardness, elasticity, and microstructure, with higher concentrations generally yielding stronger, more stable, and more printable gels, though excessive amounts may cause aggregation or inhomogeneity [107,108]. Metal ion concentration (e.g., Na^+^, K^+^, Ca^2+^, Al^3+^) similarly tunes gel microstructure and mechanical properties, with low concentrations (<0.1 M) favoring tighter, more homogeneous networks [109].

#### 4.1.2. pH and Ionic Strength

Adjusting pH and ionic strength can control gel network density, mechanical strength, and nanostructure uniformity [113,114,115]. In kidney bean protein isolate sodium alginate gels, moderate acidification and higher Ca^2+^ concentrations led to improved gel properties and a honeycomb-like structure [114]. In wheat protein nanoparticle-stabilized emulsion gels, pH near the isoelectric point (~6.8) increased aggregation and viscosity, while higher salt concentrations further enhanced viscosity and elastic modulus, allowing for the design of stable, viscoelastic gels [116]. For sodium caseinate nanoparticles, higher ionic strength during enzymatic cross-linking produced larger particles, resulting in stiffer acid-induced gels with reduced syneresis [113]. Alginate–montmorillonite nanocomposite hydrogels maintained integrity across a wide pH range, with elastic moduli increasing linearly with clay content due to physical bonds, and thinner fibers forming at extreme pH values due to increased surface charge (Figure 5B) [110,117]. In pigeon pea protein gels, low pH and low ionic strength produced coarse networks with poor water-holding, while higher pH and ionic strength yielded compact, homogeneous matrices with better water retention, highlighting the importance of hydrogen bonding and electrostatic interactions in gel formation [115].

#### 4.1.3. Co-Solutes/Sugars

In agarose-based fluid gels, increasing sucrose concentration alters the size, shape, and connectivity of microgel particles, directly impacting the gel’s viscoelasticity, texture, and lubrication by modifying the molecular interactions during gelation and shear processes [118]. For low methoxyl pectin gels, the addition of sucrose or erythritol enhances gel hardness, accelerates structuring, and increases cross-linking density, as confirmed by SEM and FTIR, with sucrose providing superior network density and physicochemical properties due to its strong hydration and chemical characteristics [119]. In gelatin-based confectionery gels, high concentrations of sweeteners raise the gelation and melting temperatures, but also weaken internal interactions and create smaller, more dispersed junction zones, while increasing gelatin concentration boosts gel hardness and tackiness [120]. For κ-carrageenan gels, sucrose as a co-solute, especially when combined with hydroxypropyl beta-cyclodextrin-curcumin composites, increases gel hardness, gumminess, and gelation temperature, and optimizes 3D printability by enhancing the gel structure formation rate [121]. Overall, sucrose’s multifaceted effects on gels stem from its ability to modulate water activity, molecular interactions, and network architecture, offering opportunities for tailored textural and functional properties in food and material applications.

#### 4.1.4. Molecular Weight and Chain Flexibility

Low-molecular-weight gelators (LMWGs) self-assemble into diverse nanostructures through non-covalent interactions, enabling morphological diversity and responsiveness to external stimuli, but often result in gels that are fragile and difficult to manipulate due to limited structural strength and flexibility (Figure 3C) [111,122]. In contrast, increasing the molecular weight (e.g., cross-linking with high-molecular-weight agents like pullulan dialdehyde) can significantly enhance the network structure, leading to improved water vapor barrier properties, reduced solubility (from 42.3% to 18.7%), and increased tensile strength (up to 2.7 times higher than neat gelatin) [123]. High-molecular-weight fractions in gelatin also yield stronger and more stable gels, though the specific effects can vary with the source and processing method [124]. Chain flexibility, influenced by the nature of the gelator and cross-linking, allows for the tuning of gel morphology and mechanical properties, as well as the ability to respond to stimuli or external shaping methods [29,125]. Moreover, the solubility and cooperative self-assembly of LMWGs are critical in determining gelation thresholds and thermal stability, highlighting the importance of molecular design in achieving desired performance.

#### 4.1.5. Blending of Components

Blending different components in food gels enables precise tuning of their properties through interactions such as covalent and non-covalent bonding, as well as co-assembly modes. For example, increasing κ-carrageenan concentration in gelatin gels accelerates gelation, enhances viscoelasticity, yield stress, and melting temperature, due to electrostatic complex formation and structural changes in the gel network [126]. Furthermore, incorporating dialdehyde κ-carrageenan into gelatin films via covalent cross-linking significantly reduced solubility, moisture content, and water vapor permeability, while increasing tensile strength by approximately 20-fold compared to pure gelatin films, and the addition of zein nanoparticles further enhanced antioxidant and antimicrobial activity when loaded with thymol [127]. In chitosan–gelatin composite films, the blending method improved elongation and swelling, while layer-by-layer assembly enhanced water vapor barrier, tensile strength, and thermal stability, demonstrating that the method of blending or assembly directly affects nanostructure and performance [128,129]. Multi-component organogels and bigels exhibit synergistic interactions between gelators, resulting in higher hardness and moduli than single-component systems, and the inclusion of colloidal particles further increases stability and mechanical strength (Figure 3D) [112]. The nanostructure and macroscopic properties of two-component dendritic gels can be modulated by adjusting component concentration, molecular structure, or molar ratio, leading to changes in gel morphology and transition temperatures [130,131,132]. Additionally, the choice of solvent in multi-component gels can drive different self-assembly modes and nanostructures, highlighting the importance of environmental factors in tuning gel properties [133].

### 4.2. Physical Control

Processing controls (e.g., temperature, shear/flow, pressure, and ultrasound) tune properties of food gels by influencing crystallization, alignment, gelation, and nano-emulsion formation.

#### 4.2.1. Temperature Control

**Figure 6:**
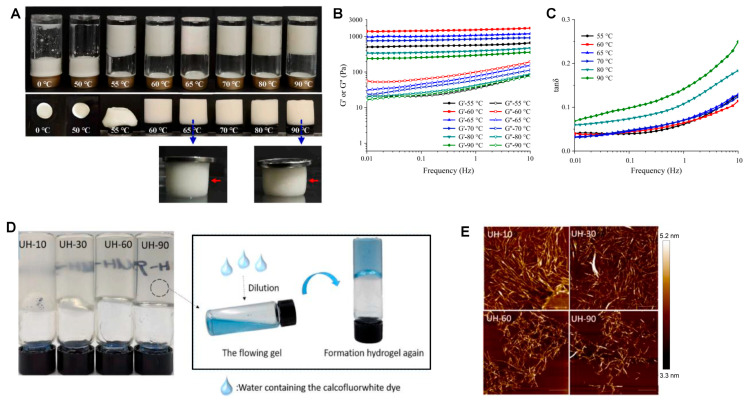
(**A**) Visual appearance of starch emulsion-filled gels formed by 0–90 °C gelation. Red arrows show the edges of the emulsion-filled gels under loading. (**B**) Storage (G’) and loss (G”) modulus, and (**C**) tan δ as a function of frequency for starch emulsion-filled gels formed by different gelation temperatures (55–90 °C) (adapted from [134] with permission from Elsevier). (**D**) The appearance of cellulose hydrogels obtained from different ultrasonic durations (left) and the reversible property of hydrogels (right). (**E**) The morphology of nanocellulose particles from different hydrogels characterized by atomic force microscopy (AFM) (adapted from [149] with permission from Elsevier). The scale is 1 μm.

In starch-based emulsion-filled gels, lower gelation temperatures (around 55 °C) promote the leaching of starch molecules, forming active networks, while moderate increases (to 60 °C) enhance gel strength due to swollen granules; however, higher temperatures can weaken the gel by causing granule disintegration, as shown in Figure 6A–C [134]. In nano-emulsion gels, sequential temperature changes can restructure the gel, allowing for precise control over microstructure and rheological properties by modulating interparticle potentials and triggering self-assembly [135,136]. For protein–starch composite gels, preheating above 60 °C alters protein conformation and promotes protein–starch complex formation, resulting in softer textures and changes in digestibility, while lower preheating temperatures yield harder, bicontinuous networks [137]. These findings demonstrate that careful temperature management enables the design of food gels with tailored nanostructures and functional properties for specific applications.

#### 4.2.2. Shear/Flow Fields

High shear rates or large strain amplitudes can fully break down gel networks, resulting in more homogeneous and stronger gels upon cessation of flow, while lower shear rates or strain amplitudes tend to create heterogeneous, weaker gels with reduced elasticity [138,139]. For example, in colloidal and flaxseed fiber gels, increasing homogenization or shear rates weakens the network structure (lower viscoelastic moduli) but can improve physical stability, making them suitable as food stabilizers [140]. Oscillatory shear is particularly effective at tuning gel properties, with large amplitudes producing single-yielding, strong gels and intermediate amplitudes leading to weak, cluster-dense structures [141]. Shear history also induces memory effects, where the microstructure and elasticity of the final gel depend on the specific shear protocol applied, such as the critical shear rate in boehmite gels that determines whether the gel exhibits glassy or gel-like viscoelastic spectra [142]. Additionally, the presence of co-solutes (e.g., sucrose) and the order of ingredient addition can further modulate the microgel network and rheological behavior under shear, impacting texture and lubrication properties [14,142].

#### 4.2.3. Pressure

Elevated pressures can lower the minimum concentration required for gel formation, as seen with pea protein and oat β-glucan gels, where higher pressures (e.g., 600 MPa for pea protein, 500 MPa for β-glucan) enable gelation at lower concentrations and enhance gel strength and textural properties [143]. Pressure also allows for the fine-tuning of gel microstructure and mechanical properties by adjusting parameters like pH, pressure level, and treatment duration, resulting in gels with diverse textures and improved water-holding or freeze-thaw stability [144]. In nano-emulsions stabilized by plant proteins, high-pressure homogenization (HPH) controls droplet size, which inversely affects gel strength (i.e., smaller droplets yield stronger gels) [145]. For gelatin, HPH disrupts hydrogen bonds and unfolds proteins, increasing emulsifying and foaming capacities but reducing gel strength and elasticity [146]. Pressure-induced gels often differ from heat-induced ones, typically exhibiting less hardness and adhesiveness but better retention of thermolabile compounds and unique textural profiles [147]. Additionally, pressure can be used to create stable Pickering emulsion gels with enhanced encapsulation and stability of bioactive compounds like curcumin [148].

#### 4.2.4. Ultrasound

Ultrasound treatment enables the fine-tuning of gel strength/hardness, microstructure, and WHC by influencing protein unfolding/aggregation and spatial distribution of other biopolymers (Figure 6D,E) [149,150]. In peanut protein systems, high-intensity ultrasound decreased nanoparticle size and enhanced heat-set gelling and emulsifying properties, especially at higher protein concentrations [151]. Ultrasound-assisted extraction of soy protein isolate led to a substantial increase in extraction yield (from 24.68% to 42.25%) and improved gel mechanical properties, uniformity, and water retention [152]. For starch-based gels, ultrasound induced cracks and pores in granules, increasing extraction yield by 14.55% and improving thermal stability and gel matrix strength [153]. In nanocellulose hydrogels, ultrasound reduced interfibrillar distances, resulting in stronger gel strength and improved emulsion stability (Figure 6D,E) [149].

**Table 1:** Summary of strategies for tuning nanostructures of gels.

Strategy	Mechanism	Examples	Impact on Nanostructure	Ref.
Concentration	Increased concentration enhances molecular proximity	Alginate gels; κ-carrageenan gels	Promotes more frequent junction zone formation, leading to denser and stronger networks	[154,155]
pH adjustment	Modulates charge distribution and gelation behavior of biopolymers	Acid/alkaline-induced casein, pea, or pectin gels	Alters network density, pore size, and aggregation behavior	[156,157,158]
Ionic strength and Ion type	Ionic cross-linking or shielding modulates gelation and structure	Ca^2+^-induced alginate gel; K^+^-induced κ-carrageenan gel; Na^+^ effect on protein gels	Controls gel stiffness, porosity, and nano-fibrillar structure	[159,160,161,162,163]
Solvent Quality/Polarity	Affects molecular interactions and phase separation	Ethanol or sugar concentration to promote gelation	Changes gel network compactness and aggregation state	[164,165,166]
Co-gelling or composite systems	Combines multiple gelling agents or nanofibrils to form hybrid structures	Alginate–gellan gum, protein–polysaccharide blends	Enhances hierarchical structure and multi-scale network architecture	[167,168,169,170,171]
Thermal treatment	Induces denaturation or conformational changes that promote gelation	Alginate gels; Heat-set whey protein gels; protein–polysaccharide gels; gelatin melting and reformation	Modulates fibril size, network junctions, and WHC	[172,173,174,175]
Shear processing	Aligns, disrupts, or restructures gel network during processing	Homogenization, extrusion, or whipping of gels	Controls anisotropy, fibrillar orientation, and nanopore structure	[176,177,178]
Pressure	Induces protein unfolding and aggregation via non-thermal means	High-pressure-treated starch or protein gels	Increases WHC and creates uniform, dense nanostructures	[143,144,147,179]
Ultrasound	Improves interaction between phases in composite gels	Protein, protein–polysaccharide, or emulsion-filled gels	Promotes finer emulsions and more uniform microphase distribution at the nanoscale	[39,180,181,182]

### 4.3. Engineering Multifunctional Gel Matrices Through Nanostructure Integration

Nanostructures (e.g., cellulose nanocrystals, protein nanofibrils, and nanoparticles) interact with gelators through covalent or non-covalent bonds, restrict water mobility, facilitate protein unfolding and cross-linking, and endow food gels with multifunctionality, like antibacterial, antioxidant, and intelligent responsiveness.

#### 4.3.1. Modulating Gel Properties via Nanoparticles as Functional Modifiers

**Figure 7:**
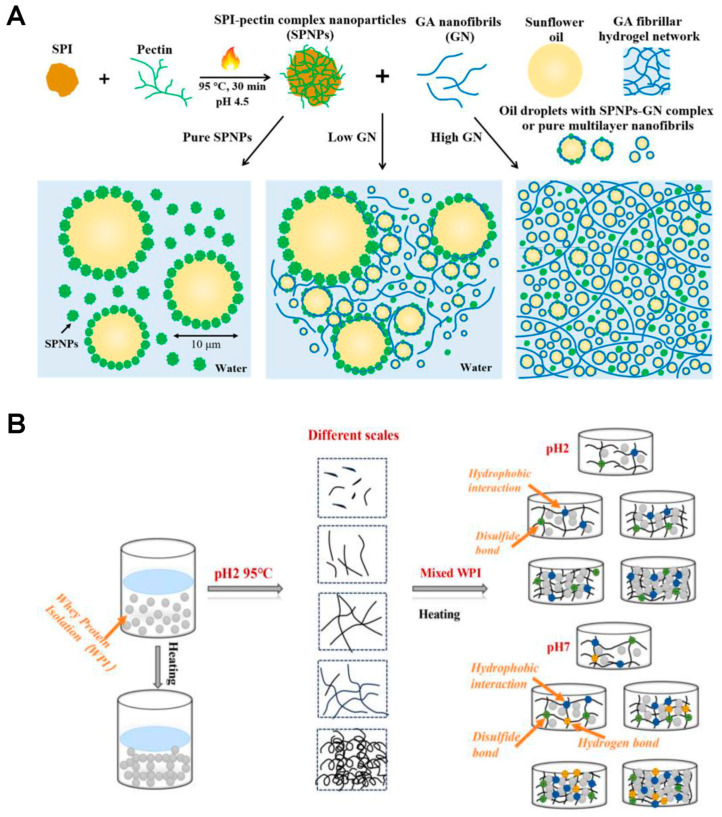
(**A**) Schematic diagram of emulsion and emulsion gels prepared by soy protein isolate (SPI)-pectin complex nanoparticles and glycyrrhizic acid (GA) nanofibrils (adapted with permission from [183]. Copyright 2020 American Chemical Society). (**B**) Mechanism diagram of whey protein isolate (WPI) composite gel strengthened by whey protein isolate fibrils (WPIF) with different scales at different pH (pH 2.0 and pH 7.0) (adapted from [189] with permission from Elsevier).

Nanoparticles tailor the properties of food gels through various mechanisms such as interfacial adsorption, network formation, and molecular interactions. For example, combining soy protein-pectin complex nanoparticles with glycyrrhizic acid nanofibrils leads to smaller emulsion droplet sizes and stronger, more thixotropic gels due to hydrogen bonding and the formation of fibrillar hydrogel networks around droplets (Figure 7A) [183]. Shellac nanoparticle–chitosan complexes, stabilized by hydrogen bonding and electrostatic interactions, serve as both emulsifiers and gelling agents, maintaining gel strength and improving bio-accessibility of encapsulated nutrients like β-carotene [184,185]. The addition of nanoparticles like NiO, ZnO, and modified κ-carrageenan also boosts the mechanical strength, barrier properties, and thermal stability of biopolymer-based films, with tensile strength increases up to 20-fold and improved antibacterial and antioxidant activities, making them ideal for active food packaging [127,186]. Starch-based and gelatin nanoparticles further contribute by enhancing film and gel stability, enabling controlled release of active ingredients, and providing responsiveness to environmental stimuli [187,188].

#### 4.3.2. Reinforcing Gel Networks Through Nanofiber-Based Structural Integration

Nanofibers (e.g., cellulose nanofibers and protein-based nanofibers) enhance the mechanical and rheological properties of food gels by reinforcing their 3D network structures and modulating interactions at the molecular level. The addition of nanofiber cellulose to gelatin gels increases hardness and viscosity, transforms rheological behavior from Newtonian to pseudoplastic, and raises the sol–gel transition temperature [108]. Whey protein nanofibers incorporated into protein gels boost gel strength, WHC, and storage modulus, while also shortening gelation time and creating highly porous microstructures (Figure 7B) [189]. The gelation of nanofiber-based gels is primarily driven by electrostatic interactions, fiber entanglement [190]. However, in some systems, ion-induced conformational changes also promote protein unfolding and aggregation into stable networks. For example, increasing calcium ions in cellulose nanocrystal–whey protein gels enables gel formation by promoting β-sheet formation and hydrophobic interactions [68]. Additionally, nanofiber incorporation can improve cold water solubility, emulsion stability, and foam-forming ability in gelatin gels, broadening their functional applications [191].

#### 4.3.3. Safety and Regulatory Concerns of Nanomaterials in Food Products

Involving nanomaterials in food gels raises significant safety and regulatory concerns due to their small size and high surface area, which enable them to cross biological barriers and potentially induce toxic effects such as oxidative stress, genotoxicity, and inflammation in human cells [192]. Migration of nanoparticles from packaging or other gel products into food is a key risk, with studies showing that materials like silver, titanium dioxide, and zinc oxide nanoparticles can leach under certain conditions, leading to human exposure [193]. Regulatory frameworks remain inconsistent globally, with some countries lacking strict controls, and there is a pressing need for comprehensive legislation and standardized risk assessment methods to ensure consumer safety [194,195]. Several reports highlight that nanoparticles fully embedded in polymer matrices are less likely to migrate [196], but surface alterations or mechanical stress can increase release, and the lack of long-term toxicity data continues to hinder regulatory progress.

## 5. Nanostructural Modulation for Function Control in Food Gels

To develop food gels for multiple applications, we need to understand how the structure at the nanoscale level influences their functionalities, which could be concluded into four aspects (i.e., mechanical/textural properties, WHC, optical properties, and mass transport/release).

### 5.1. Texture Customization Through Controlled Mechanics of Nanostructures

The mechanical and textural properties of food gels, such as gel strength, elasticity, adhesiveness, and breakdown behavior, are crucial for food quality and sensory perception [15,197]. Nanostructures modulate these properties by reinforcing gel matrices, improving elasticity, ductility, and water resistance. For example, incorporating nanofibers or nanoparticles such as ZnO into biopolymer-based gels increases Young’s modulus, tensile strength, and elongation at break, resulting in stronger gels suitable for food packaging and high-moisture foods [198,199]. Oxidized cellulose nanofibrils, even at low concentrations, synergistically improve surimi gel whiteness, gel strength, and thermal stability through matrix reinforcement and water binding [200]. The formation of rod-like junction zones and point-like cross-links in pectin-calcium gels directly influences their mechanical strength and swelling behavior [83]. Similarly, protein-based nanostructures and nano-emulsion-based gels allow for tailored texture, such as consistency and shear-thinning behavior, by modulating the type and concentration of gelling agents [90,201]. These advances are particularly valuable for developing foods with specific textural requirements, such as those for the senior population or individuals with dysphagia, where both safety and palatability are critical.

### 5.2. Controlling WHC and Stability via Nanostructure-Mediated Water Confinement

Nanostructures (usually hydrophilic cellulose and protein nanofibers) can reinforce gel networks, promote water entrapment, improve gel strength and viscoelasticity, and are essential for WHC and stability of food gels. Mechanistically, these nanostructures act as active fillers, restrict water mobility, induce favorable protein conformational changes, and strengthen the gel matrix via hydrophobic, hydrogen, and disulfide bonds [103,189]. For example, adding cellulose nanocrystals to whey protein isolate gels increased WHC, gel strength, and thermal stability by facilitating protein unfolding, cross-linking, and forming compact, homogeneous structures, with WHC rising as CNC concentration increased up to 1% *w*/*v* [103]. Similarly, oxidized cellulose nanofibrils at low concentrations greatly improved surimi gel WHC through matrix reinforcement, water binding [200]. However, in Pickering emulsion gels, gelatin or xanthan gum/lysozyme nanoparticles also increased WHC by enhancing network density and droplet surface coverage [202].

### 5.3. Modulating Light Transmission, Scattering, and Absorption via Nanostructured Matrices

Optical properties of food gels can be influenced by various factors, like compositions, structures, and environmental conditions, enabling improvement in food packaging and sensory perception. The size and distribution of nanostructures within gels influence light scattering, affecting transparency, opacity, and color. For example, incorporation of Co-doped ZnO nanoparticles into chitosan/gelatin gels reduces the optical energy bandgap (from 5.22 to 4.90 eV direct, and 4.92 to 4.42 eV indirect), enhancing optical, dielectric, and antimicrobial properties [203]. Additionally, the supramolecular ordering and chiral nano-structuring in chitosan-aspartate glycerohydrogels result in wavelength-dependent transmittance and scattering, and the novel effect of optical clearing in the near-UV region, which is influenced by the size and shape of nanodomains [204]. Moreover, nanostructural inhomogeneity in polymer network gels, often arising from variations in polymer-segmental and cross-linking densities, affects optical clarity and performance, with more homogeneous nanostructures leading to improved transparency and functional properties [205].

### 5.4. Programming Bioactive Release Through Engineered Nanostructural Barriers/Diffusion

Controlling the oral delivery and bioavailability of active compounds is a promising function of food gels and also a hotspot recently, which emphasizes the health and wellness properties of products [206]. Nanostructures (e.g., cellulose nanocrystals, protein nanoparticles, and nanofibrils) enhance this function by modifying gel microstructure, increasing gel strength, and improving WHC [103,207]. However, it remains unclear how gel strength and WHC of various structures are mechanistically related to the controlled release of encapsulated compounds. For example, protein nanostructures and nanohydrogels (e.g., lactoferrin nanohydrogels) provide high encapsulation efficiency (up to 90%) and allow for controlled, matrix-dependent release of bioactives like curcumin, with release rates varying based on the hydrophilic or lipophilic nature of the surrounding medium [90,206]. Microemulsions with nanostructured matrices also show that changes in viscosity and microstructure can shift release kinetics from rapid to sustained, with systems containing thickeners like carboxymethyl cellulose exhibiting matrix-driven, slower release profiles [208].

## 6. Applications Enabled by Tuning Nanostructure of Food Gels

**Figure 8:**
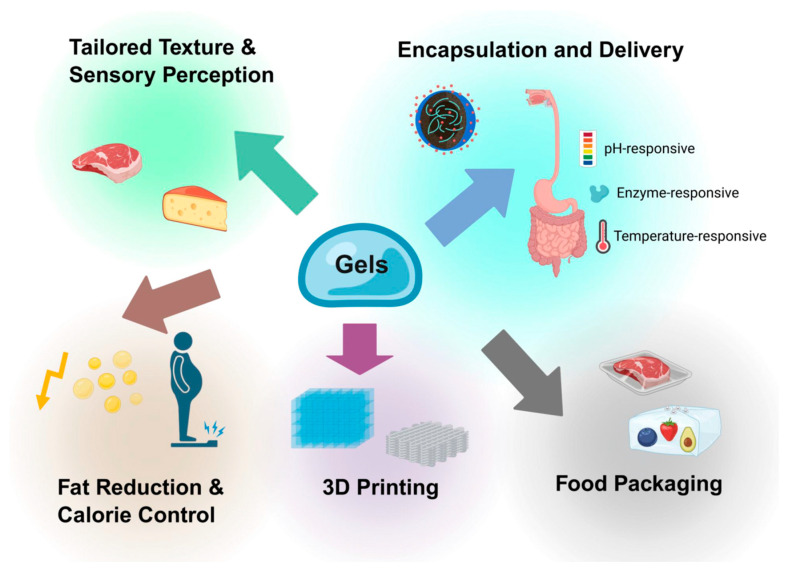
Schematic diagram of various applications of gels in food areas.

Tuning nanostructures of food gels has emerged as a powerful tool to achieve specific applications, such as tailored texture, controlled release, 3D/4D printing adaptation, fat reduction, smart packaging solutions, etc., as shown in Figure 8. The successfully developed applications of food gels are summarized in Table 2.

### 6.1. Tailored Texture and Sensory Perception

Recent works have demonstrated that tuning the nano-/microstructure of gels, through ingredient selection, processing, and the use of biopolymers or nanofibers, enables precise control over texture and sensory perception. For example, combining plant proteins with various polysaccharides (e.g., gellan gum, carrageenan, and pectin) allows the creation of gels with customizable firmness, spreadability, and crumbliness, suitable for applications like meat analogs and cheeses [228]. However, the addition of nanofiber cellulose to gelatin gels reduces elasticity and also alters rheological properties such as viscosity and sol–gel transition temperature [108]. Emulsion gels, which incorporate dispersed phases into gel matrices, offer further flexibility in designing breakdown behavior and sensory attributes, supporting applications in fat replacement [2]. Additionally, the interplay between texture and flavor/aroma release is significant: hydrocolloid-based texture modifications can alter flavor release profiles, while specific aromas (e.g., limonene, menthol) can modulate perceived firmness, stickiness, and creaminess [229].

### 6.2. Encapsulation and Delivery of Bioactives

To apply food gels as encapsulation and delivery vehicles for bioactives, recent advances have focused on optimizing gels’ nanostructures, such as nanoorganogels, nanostructured proteins, lipid-based nanocarriers, and microgels. Nanoorganogels, for example, improve the bioavailability and shelf-life of nutraceuticals by preventing degradation and enabling controlled release [230]. Nanoparticles, nanogels, and nanofibers offer pH-responsive and tunable release profiles, with encapsulation efficiency and release behavior dependent on nanostructure type and physiological conditions [231]. Furthermore, to deliver multiple bioactives, co-encapsulation systems and Pickering emulsions have been developed using protein, polysaccharide, or lipid-based assemblies to accommodate compounds with varying solubility [185,232]. Microfluidic fabrication of microgels also allows precise control over gel size and encapsulation efficiency (30–40%), enabling prolonged release and low cytotoxicity for food and pharmaceutical applications [233]. Summarily, the integration of advanced gel nanostructures, co-encapsulation strategies, and microfluidic technologies is driving the development of next-generation food delivery systems, with a focus on enhancing bioactive stability, targeted release, and functional food innovation.

### 6.3. Fat Reduction and Calorie Control

Food gels are effective fat replacers for calorie control, and modifying their nanostructure helps achieve optimal sensory and functional qualities. For example, composite gels made from citrus insoluble nanofibers and amylose at a 1:4 ratio closely mimic the creamy texture of fat and significantly inhibit lipid digestion [234]. Similarly, low-fat Pickering emulsion gels stabilized by zein/phytic acid nanoparticles form dense barriers around oil droplets, preventing coalescence and improving the stability of encapsulated curcumin [235]. Other innovations include composite gels made from konjac glucomannan and nano-citrus fiber, which can replicate the texture and stability of whipped animal cream [236]. Additionally, nanocellulose additives have been shown to reduce triglyceride hydrolysis by up to 48.4% in vitro and lower postprandial serum triglycerides by 36% in vivo, primarily by reducing lipase access and sequestering bile salts [237].

### 6.4. Food Packaging with Improved Shelf-Life and Smart Responsiveness

Precisely tuning the nanostructures of food gels enhances gel-based packaging performance, such as extended food shelf-life, smart freshness, and microbiological detection. This improvement is consistently achieved by incorporating nanomaterials like metal nanoparticles, nanofibers, or nanohybrids, which enhance antimicrobial activity, mechanical strength, and smart responsiveness. Key examples include: gelatin-based films incorporating zinc metal-organic frameworks (Zn-MOFs) achieved up to 100% antioxidant activity and exhibited strong antibacterial effects, significantly delaying texture deterioration and inhibiting microbial growth in cherry tomatoes during 16 days of storage [238]. Similarly, nano-emulsions incorporated into packaging systems have been shown to prevent microbial growth, reduce oxidation, and browning [239]. Moreover, tuning nanostructures not only improves mechanical and barrier properties but also enables real-time monitoring of food quality through smart features like sensors and pH-responsive tags, which can indicate spoilage or freshness [240,241]. Usually, these innovations, often based on biopolymers and sustainable materials, integrate antioxidant, antimicrobial, and freshness-indicating properties, although challenges remain regarding scalability, cost, and regulatory acceptance [21,242]. Moreover, the balance between nanostructure functionality and safety must be carefully managed, with future research needed to address long-term health and environmental impacts.

### 6.5. Novel Food Structures and 3D Printing

By optimizing the rheological and mechanical properties of gels (e.g., starch, protein, and Pickering emulsions), researchers have achieved improved shape accuracy, structural integrity, and sensory qualities in printed foods, as seen with sorghum protein gels at 25% concentration and optimized nozzle size [243,244]. The incorporation of innovative additives like lipids and carbohydrates further tailors gel properties, improving their printability and sensory attributes [245]. Strategies such as reinforcing emulsion gel interfaces, cross-linking, and employing 3D or coaxial printing for pH-responsive hydrogels have enabled the creation of personalized, texture-modified foods tailored for individuals with dysphagia or specific dietary needs, improving both nutritional value and swallowing safety [223,246]. Moreover, the interaction between gel components and environmental factors (e.g., ionic strength, temperature, pH) has been shown to influence the final 3D print precision, as demonstrated by the enhancement of cassava starch gel with casein protein, which increased molecular weight and elastic modulus, leading to higher printing accuracy [247]. Summarily, the predictability of print ability can be quantitatively linked to specific rheological parameters, such as phase angle and gel network density, allowing for more rational design of food inks.

## 7. Challenges and Perspectives

### 7.1. Current Challenges

Owing to their tunable properties and compatibility with natural ingredients, food gels find wide application in texture design, fat replacement, nutrient delivery, 3D printing, and food packaging. Nanostructure tuning is a crucial strategy for precisely optimizing gel properties, enabling the development of gels with tailored properties. However, challenges remain, including the complexity of multicomponent systems, difficulties in achieving precise structural control and predictability, and the need to balance mechanical performance with durability and stability. Additionally, integrating diverse functional nanomaterials without compromising gel integrity, scaling up manufacturing processes, and meeting application-specific requirements such as biocompatibility and environmental adaptability remain major hurdles. The addition of solid nanoparticles (e.g., glass, titanium dioxide) to food gels also may pose health risks by inducing toxicity, disrupting nutrient absorption, or causing oxidative stress, especially if their interactions with food components are not well understood or controlled. These issues are compounded by limited understanding of nanostructure–property relationships and evolving safety and regulatory concerns, underscoring the necessity for continued research to fully realize the potential of tuning nanostructures of food gels.

### 7.2. Future Directions

The future of food gels is marked by rapid advancements in nanostructure tuning technologies. Emerging trends include the use of natural, plant-based nanomaterials, double-network and hybrid gel systems, and 3D/4D printing for personalized nutrition and food design. Challenges remain in scaling up production, ensuring safety and consumer acceptance, and fully understanding the complex interactions at the nanoscale, but ongoing research is expected to yield sustainable, multifunctional food gels with tailored properties for health, sensory, and environmental benefits. Future research is expected to solve the following open research questions: (1) How can nanostructured food gels be safely scaled up for industrial production? (2) What are the long-term health and safety implications of consuming nanostructured food gels? (3) How can 3D/4D printing and plant-based nanomaterials be optimized for personalized nutrition? In summary, tuning food gels’ nanostructure is poised to revolutionize food design for multiple applications, but further research is needed to address key challenges and unlock their full potential.

## 8. Conclusions

This review has examined the significance of nanostructures in food gels, emphasizing how precise tuning can lead to enhanced properties and novel applications in the food industry. By manipulating the building blocks, gelation mechanisms, and processing conditions, researchers can control the nanoscale architecture of food gels, thereby tailoring their mechanical, textural, and functional characteristics. The integration of advanced characterization techniques has been pivotal in understanding and optimizing these nanostructures, enabling the development of gels with specific attributes such as improved WHC, controlled release of bioactives, and customized sensory profiles. Applications ranging from texture modification and fat reduction to 3D food printing and smart pack aging illustrate the versatility and potential of nanostructured food gels. However, several challenges persist, including the complexity of multicomponent systems, the need for precise structural control, and the scalability of production processes. Additionally, ensuring the safety and regulatory compliance of nanostructured materials in food products remains a critical concern. Future research should focus on overcoming these challenges by developing innovative methods for nanostructure manipulation, enhancing the understanding of structure-property relationships, and exploring sustainable and biocompatible materials. Interdisciplinary collaboration will be essential in driving forward the field, fostering the creation of next-generation food products that meet consumer demands for quality, health, and sustainability. In conclusion, tuning the nanostructures of food gels represents a frontier in food science and engineering, offering unprecedented opportunities for innovation and improvement in food design and functionality. Continued exploration and advancement in this area will undoubtedly contribute to the evolution of the food industry, addressing both current and future challenges.

## Figures and Tables

**Table 2:** Summary of gels’ food applications.

Application	Function	Examples	Key Benefits	Ref.
Textural modification	Modify mouthfeel, texture, and rheology	Restructured meat, plant-based foods	Improves sensory appeal and consumer acceptability	[45,209,210]
Fat replacement	Mimic fat mouthfeel with lower calorie content	Protein or polysaccharide-based gels fat mimetics	Reduces calorie content while maintaining creamy texture	[211,212,213,214]
Flavor encapsulation	Trap and release volatile aroma/flavor compounds	Emulsion-filled gels, hydrogels with flavor compounds	Controlled flavor release and protection from oxidation	[215,216,217]
Nutrient delivery	Controlled release and protection of bioactives	Gels encapsulating vitamins, probiotics, polyphenols	Enhances stability, bioavailability, and targeted release	[218,219,220]
Satiety enhancement	Induce gastric retention or swelling to promote fullness	β-Glucan, or protein gels for appetite regulation	Supports weight management and satiety	[221,222]
3D food printing	Serve as printable bio-ink or scaffold for customized food shapes	Alginate, pectin, starch, or protein-based printable gels	Enables designable textures and personalized nutrition	[223,224]
Edible coatings/films	Serve as sustainable food packaging materials	Gelatin or alginate-based films on fruits or meat	Enhances shelf-life and appearance	[225,226]
Water or oil structuring	Structure liquids into gels for functional or sensory improvement	Oleogels, hydrogel particles in beverages	Stabilizes emulsions and improves mouthfeel in reduced-fat products	[227]

## Data Availability

Not applicable.

## Abbreviations

The following abbreviations are used in this manuscript:

WHC	Water-Holding Capacity
3D	Three-Dimensional
IDDSI	International Dysphagia Diet Standardization Initiative
Cryo-SEM	Cryo-Scanning Electron Microscopy
TEM	Transmission Electron Microscopy
AFM	Atomic Force Microscopy
STED	Stimulated Emission Depletion
FLIM	Fluorescence Lifetime Imaging Microscopy
LM	Light Microscopy
PLM	Polarized Light Microscopy
SAXS	Small-Angle X-Ray Scattering
SANS	Small-Angle Neutron Scattering
USANS	Ultra-Small-Angle Neutron Scattering
FTIR	Fourier-Transform Infrared Spectroscopy
NMR	Nuclear Magnetic Resonance
LMWGs	Low-Molecular-Weight Gelators
ICP-ASE	Inductively Coupled Plasma Atomic Emission Spectroscopy
CNC	Cellulose Nanocrystals
HPH	High-Pressure Homogenization
